# Exploring the Evolutionary History of the Differentially Expressed Genes between Human Populations: Action of Recent Positive Selection

**DOI:** 10.4137/ebo.s744

**Published:** 2008-05-15

**Authors:** Wei Zhang, M. Eileen Dolan

**Affiliations:** 1 Section of Hematology/Oncology, Department of Medicine; 2 Committee on Clinical Pharmacology and Pharmacogenomics; 3 Cancer Research Center, The University of Chicago, IL 60637, U.S.A

**Keywords:** gene expression, recent positive selection, population difference, human evolution

## Abstract

Though debates exist on the early human evolutionary models such as the “Out of Africa” theory, which hypothesizes that modern humans migrated from Africa to Europe about 50,000 to 100,000 years ago, Africans and Europeans were geographically separated with minimal gene flow for tens of thousands of years. The variations between the current European and African populations, therefore, should have evolved during this timeframe. To gain more insights into the evolutionary history of human phenotypes including gene expression, it is critical to tell how recent positive selection has played a role in the variations observed in the current populations. Using the list of differentially expressed genes we previously identified between the HapMap samples derived from individuals of African (from Ibadan, Nigeria) and European (from Utah, USA) ancestry, we searched for evidence of selection among these differential genes. We found that 27 differentially expressed genes (out of 356 tested) between these two European and African populations have been under recent positive selection. Our findings suggest that the variation between these two populations appears to be affected primarily by neutral genetic drift and/or stabilizing selection and to a lesser degree by positive selection. Further annotation enrichment analyses showed that these 27 genes under selection were overrepresented in certain Gene Ontology biological processes, molecular functions and cellular components such as transcription, lipid binding and lysosome. Our results can provide unique insights into the evolutionary history of the variation in the gene expression phenotype between these two human populations.

## Introduction

The early history of human evolution is still uncertain, though a popularly-called “Out of Africa” model hypothesizes that behaviorally modern humans (*Homo sapiens sapiens*) evolved in Africa 200,000 years ago and migrated northward from Africa to Europe, then spread to other parts of the world in the past 50,000 to 100,000 years (Stinger, 1974a; Stringer and Andrews, 1988). Analysis of mitochondrial DNAs demonstrated evidence that geographical populations including Africans and Caucasians stemmed from one woman who was postulated to have lived about 200,000 years ago, probably in Africa (Cann et al. 1987; Wills, 1992). Based on the history of global climate changes, a recent study suggested that recovery from a megadrought 100,000 years ago precipitated the exodus of ancient humans from tropical Africa to Europe and elsewhere (Cohen et al. 2007). However, no matter this or other competing models reflect the true evolutionary human history or not, the fact is that Africans and Europeans were geographically separated with minimal gene flow for tens of thousands of years (Templeton, 2005). Therefore, the phenotypes and genotypes of the human populations derived from African and European ancestry should have differentiated during this timeframe. Theoretically, the genetic variations among current populations could be due to mechanisms such as genetic drift and/or natural selection. On one hand, each of the dramatic changes including the potentially challenging new environments, global climate changes (e.g. the last ice age ended 14,000 years ago), the transition from hunter-gatherer to agricultural societies as well as the rapid increases in human densities could have resulted in powerful selective pressures for new genotypes that were better suited for the habitats. On the other hand, since during the majority of the last 100,000 years human population densities were so sparse (Casteel, 1972) that random mechanisms such as genetic drift could also have worked to cause genetic variations in the current populations. However, phenotypic variation (due to underlying heritable genetic variation and other non-genetic factors) is a fundamental prerequisite for evolution by natural selection (Wagner and Altenberg, 1995). Without phenotypic variation, there would be no evolution by natural selection. To understand the evolutionary history of human variations, it is critical to estimate how selection has played a role in the observed phenotypic variations among current populations. Numerous studies have identified and quantified signatures of selection in the human genome and human phenotypic variation (Bamshad and Wooding, 2003; Nielsen, 2005). For example, signatures of positive selection in genes associated with human skin pigmentation have been revealed from analyses of single nucleotide polymorphisms (SNPs) (McEvoy et al. 2006; Lao et al. 2007). Other examples of signatures of selection include genes related to the categories such as chemosensory perception (e.g. bitter-taste) and olfaction as well as gametogenesis, spermatogenesis, and fertilization (Soranzo et al. 2005; Voight et al. 2006). Since gene expression as a phenotype is a complex quantitative trait partially regulated by genetic variation in DNA sequence, we therefore searched for genes that have been the targets of recent positive selection among a list of differentially expressed genes between two populations of African and European descendents to illustrate the evolutionary history of this particular phenotypic variation (i.e. gene expression).

Using the lymphoblastoid cell lines (LCLs) from the International HapMap Project (International HapMap Consortium 2003; International HapMap Consortium 2005), gene expression as a phenotype as well as its variation between individuals of African (YRI: Yoruba people from Ibadan, Nigeria) and European (CEU: Caucasian people from Utah, USA) ancestry has been investigated (Storey et al. 2007; Zhang et al. 2008a; Zhang et al. 2008b). By characterizing patterns of natural expression variation in 16 individuals from the CEU and YRI samples, Storey et al. found extensive variation in gene expression levels (Storey et al. 2007). Particularly, we identified differences in 383 gene expression phenotypes between the CEU and YRI samples and evaluated the contribution of common genetic variants to theses population differences (Zhang et al. 2008a). Using the unrelated HapMap LCLs including 60 CEU and 60 YRI samples, Voight et al. described an analytical method for scanning SNPs for signals of recent positive selection (Voight et al. 2006). Integrated haplotype score (iHS), which measures the possibility that a gene has undergone recent positive selection was developed to detect evidence of recent positive selection at a locus. It is based on the differential levels of linkage disequilibrium surrounding a positively selected allele compared to the background allele at the same position (Voight et al. 2006). We then used the web application Happlotter (http://hg-wen.uchicago.edu/selection/) (Voight et al. 2006) to find genes with strong signals of selection among our list of differentially expressed genes between the CEU and YRI samples. Furthermore, we performed annotation enrichment analyses among the genes under selection using the Gene Ontology (GO) (Ashburner et al. 2000) and Kyoto Encyclopedia of Genes and Genomes (KEGG) (Kanehisa and Goto, 2000; Kanehisa et al. 2006; Kanehisa et al. 2007) databases.

## Materials and Methods

### Differentially-expressed genes between human populations

Gene expression in 176 HapMap LCLs (87 CEU and 89 YRI samples) for ~17,500 transcript clusters (gene-level) had been evaluated using the Affymetrix GeneChip^®^ Human Exon 1.0 ST Array (Affymetrix, Inc., Santa Clara, CA, U.S.A). Gene-level expression of transcript clusters was summarized using the robust multi-array average (RMA) (Irizarry et al. 2003) method with signals generated on a core set (i.e. with RefSeq supported annotation) (Pruitt et al. 2005; Pruitt et al. 2007) of exons and deposited at Gene Expression Omnibus (GEO, http://www.ncbi.nlm.nih.gov/projects/geo/) (GEO accession: GSE7851). Gene expression was found to differ significantly between the CEU and YRI samples for 383 transcript clusters (Zhang et al. 2008a) among ~9,200 expressed transcript clusters in LCLs. To avoid identity ambiguity, our analysis set of differentially-expressed genes is comprised of 356 differential transcript clusters with unique annotation provided by the Affymetrix NetAffx Analysis Center website (http://www.affymetrix.com/analysis/index.affx). These genes have a one-to-one relationship between the Affymetrix transcript cluster ID and gene annotation (NCBI B36 Assembly, March, 2006).

### Evidence of recent positive selection

Happlotter (http://hg-wen.uchicago.edu/selection/) (Voight et al. 2006) was used to check if a particular gene has been a target of recent positive selection. Happlotter is a web application that has been developed to display the results of a scan for positive selection in the human genome using the HapMap data. The current version contains results using the Phase II data (~3.1 million SNPs, HapMap release 21) of the HapMap Project (International HapMap Consortium, 2005; Frazer et al. 2007). Specifically, we used the Happlotter-calculated iHS to measure the possibility of a gene that has undergone recent positive selection. The empirical p values, quantified by the proportion of SNPs with |iHS| >2 for each bin of 50 neighboring SNPs, were generated by Happlotter (Voight et al. 2006). Simulations indicated that this criterion provides a powerful signal of selection (Voight et al. 2006). The empirical p value of 0.05 was used as the cutoff for significance.

### Gene ontology and pathway databases

We searched the GO (http://www.geneontology.org/) (Ashburner et al. 2000) and KEGG (http://www.genome.jp/kegg/) (Kanehisa and Goto, 2000; Kanehisa et al. 2006; Kanehisa et al. 2007) databases to more thoroughly understand the functions of those genes that have evidence for recent positive selection. The three organizing principles of GO (database release March, 2008) are molecular function, biological process and cellular component. A gene product has one or more molecular functions and is used in one or more biological processes; it might be associated with one or more cellular components. KEGG is a collection of manually drawn pathway maps representing the current knowledge on the molecular interaction and reaction networks for metabolism, genetic information processing, environmental information processing, cellular processes and human diseases. The current version contains 354 reference pathways (release 45.0, January 1, 2008). Onto-Express (Draghici et al. 2003a; Draghici et al. 2003b; Khatri et al. 2004) was used to identify any enriched GO terms in the genes under selection relative to all the differentially expressed genes. Similarly, Pathway-Express (Draghici et al. 2003a; Draghici et al. 2003b; Khatri et al. 2004) was used to identify any enriched KEGG pathways. A false discovery rate (FDR) of 20% (corrected p value) after Benjamini-Hochberg (BH) correction (Benjamini and Hochberg, 1995) was used for significance in these enrichment analyses (with at least 2 genes).

## Results

### Differentially expressed genes under recent positive selection

**Figure 1:**
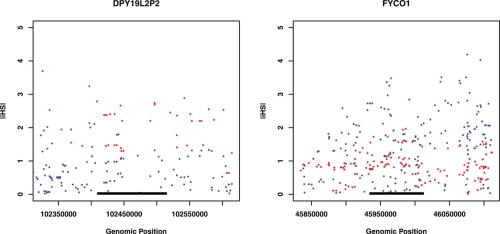
Two genes with expression higher in CEU are under recent positive selection. (A) *DPY19L2P2* (Chr7: 102,409,531–102,514,791) is under selection in CEU (Red); (B) *FYCO1* (Chr3: 45,934,399–46,012,303) is under selection in YRI (Blue). X-axis is genomic position (HapMap release 21, dbSNP b125). Y-axis is |iHS| score. The |iHS| cutoff for selection is 2. The target gene is marked by a black bar. The 100 Kb flanking regions are also showed.

**Figure 2:**
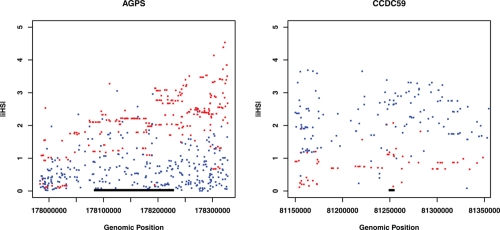
Two genes with expression higher in YRI are under recent positive selection. (A) *AGPS* (Chr2: 178,083,009 –178,228,511) is under selection in CEU (Red); (B) *CCDC59* (Chr12: 81,249,086–81,254,640) is under selection in YRI (Blue). X-axis is genomic position (HapMap release 21, dbSNP b125). Y-axis is |iHS| score. The |iHS| cutoff for selection is 2. The target gene is marked by a black bar. The 100 Kb flanking regions are also showed.

Using the web application Happlotter, 27 genes were found to be targets of significant recent positive selection (empirical *P* < 0.05) (Table 1). Among them, 26 genes are under selection in either CEU or YRI samples (13 in CEU and 13 in YRI). One gene, *HNRPH3*, is under selection in both populations. Considering gene expression and the selection profile, there could be 4 combinations: 1) 8 genes with higher expression in YRI are under selection in YRI only; 2) 6 genes with higher expression in YRI are under selection in CEU only; 3) 7 genes with higher expression in CEU are under selection in CEU only; and 4) 5 genes with higher expression in CEU are under selection in YRI only. Some examples are shown in Figures 1 and 2. In contrast, expression *HNRPH3* was found to be higher in YRI. Supplemental Tables 1 and 2 list the data for all differential genes we tested.

### Gene ontology and pathway analyses of the genes under selection

Table 2 shows the enriched GO terms among the 27 differential genes under selection relative to the background of all differential genes. At FDR<20%, three biological processes (transcription, regulation of transcription from RNA polymerase II promoter and apoptosis), four molecular functions (lipid binding, binding, metal ion binding, transcription factor activity) and 2 cellular components (lysosome, mitochondrion) were overrepresented. At FDR<20%, no KEGG pathway was enriched among these genes.

## Discussion

Though debates exist on the models of human evolution such as the “Out of Africa” theory, which hypothesizes that modern humans in Europe migrated from Africa about 50,000 to 100,000 years ago (Stinger, 1974b; Stringer and Andrews, 1988), the variations between the current European and African populations, however, should have evolved during this timeframe. In this work, we focused on the evolutionary history of the variation in one important phenotype, gene expression, between individuals of European and African ancestry.

Previously, we identified a list of differentially expressed genes between the HapMap CEU and YRI samples (Zhang et al. 2008a). Our first goal was to identify those differential genes that have been the targets of significant recent positive selection. Using Happlotter, 27 genes out of the total 356 differential genes in the analysis set showed strong signals for selection (empirical *P* < 0.05) (Table 1). Most of these 27 genes have selectively lower or selectively higher expression in either CEU or YRI samples. Only one gene, *HNRPH3* (with higher expression in YRI) has evidence of selection in both populations. *HNRPH3* (heterogeneous nuclear ribonucleoprotein H3) is involved in the biological process of nuclear RNA splicing (Ashburner et al. 2000; Honore, 2000). This suggests its higher expression in YRI and lower expression in CEU may have certain evolutionary advantages in each population (potentially) through the regulation of alternative splicing. The differences in alternative splicing or transcript isoform variation between these populations, however, havenotbeencomprehensively investigated yet (Zhang et al. 2008b).

To further illustrate the functions of these genes, our next goal was to investigate if the 27 differential genes under selection were enriched in certain specific GO terms and/or known pathways. Interestingly, many enriched biological processes and molecular functions are related to transcription and its regulation (Table 2). For example, one enriched biological process: transcription is comprised of six genes under selection (*CCDC59*, *VPS36*, *TCF7*, *ZKSCAN5*, *IKZF3* and *HDAC1*), among which three (*ZKSCAN5*, *IKZF3* and *HDAC1*) are involved in an enriched molecular function: transcription factor activity. Clearly, the selection of these transcription-related genes can influence the phenotypes of more downstream genes, which may not be the direct targets for selection themselves. The most significant enrichment of the GO terms is the molecular function of lipid binding (*P* = 0.0056, Corrected *P* = 0.032), involving two genes (*VPS36* and *DPP7*) (Table 2). In contrast, Voight et al. showed that lipid and fatty acid binding was enriched among all of the genes with evidence for partial sweeps in one or more populations (Voight et al. 2006). *VPS36* (vacuolar protein sorting 36 homolog, *S. cerevisiae*) is involved in the enriched biological process of transcription, suggesting its role in the regulation of transcription. It is also in the enriched cellular component of lysosome, indicating its role in macromolecule digestion. Though the function of *VPS36* in humans is not clear, its homolog in yeast has been shown to be involved in the negative regulation of transcription from RNA polymerase II promoter by glucose (Kamura et al. 2001). Therefore, its selection in YRI (with higher expression) might be due to the differences in food sources between the European and African populations. In contrast, *DPP7* (phosphatidylcholine transfer protein) is involved in lipid transport (Cohen et al. 1999) through the activity of phosphatidylcholine transmembrane transporter (Cohen et al. 1999; van Helvoort et al. 1999). Potentially, this suggests that its selectively lower expression in YRI might also be related to the differences in food sources, which are different between sub-Sahara Africans and Europeans. Notable, the typical Yoruba diet has been heavy with starchy tubers, fruits and grains, light on animal foods (Bascom, 1951) while the northern European diet generally consists of a large serving of meat, poultry, or fish, accompanied by small side dishes of vegetables and starch (James, 2004). Though an important type of selective pressure that has confronted modern humans is the transition to novel food sources with the advent of agriculture, domestication and the colonization of new habitats, we still lack many details (e.g. the history of food-source changes and biological function of these genes) to link these together to interpret the selection signatures we observed. The most widely-appreciated example in this category is probably the gene encoding lactase (*LCT*) which is essential for digestive hydrolysis of lactose in milk. Lactase persistence is associated with one haplotype, which is very common only in northern Europeans (Harvey et al. 1998; Hollox et al. 2001). Evidence has shown that early Europeans were unable to digest milk because the gene *LCT* was missing in Neolithic skeletons dating to between 5,840 and 5,000 BC, therefore, the ability to drink milk was thought to be the most advantageous trait that was evolved in Europeans in the recent past (Burger et al. 2007). Interestingly, convergent evolution of *LCT* due to strong selective pressure resulting from shared cultural traits (i.e. animal domestication and adult milk consumption) has been observed in certain African and European populations (Tishkoff et al. 2007). On one hand, the fact that signals of selection around genes involved in the metabolism of carbohydrates, fat and alcohol have been found to be enriched in these populations (Voight et al. 2006) lends support to the idea of dietary adaptations in terms of processing new food sources during human evolution. On the other hand, our results further suggest that the selection due to dietary adaptations could play a role in defining at least some differential gene expression between the current CEU and YRI populations.

Overall, we found a small fraction (~8%) of the differentially expressed genes between the CEU and YRI samples that have been under strong recent positive selection. Therefore, the majority of the differential genes do not have evidence for positive selection based on the current data, suggesting other mechanisms (e.g. genetic drift, stabilizing selection) could be responsible for their variations among the current European and African populations. Previous studies in other organisms have also suggested that divergence among populations is primarily affected by neutral drift and stabilizing selection and to a lesser degree by directional selection (Whitehead and Crawford, 2006). A recent study uisng the HapMap genotypic data showed that negative selection has globally reduced human population differentiation at amino acid-altering mutations, particularly in disease-related genes, while positive selection has ensured the regional adaptation of human populations by increasing population differentiation in gene regions (Barreiro et al. 2008). However, since the HapMap genotypic data may not be able to capture all genetic variations among these populations (Tantoso et al. 2006; Zhang and Dolan, 2008b), we can not rule out the possibility that we could have missed some signals of selection because of the untyped and undiscovered SNPs. The results from the deep resequencing projects such as the SeattleSNPs Project (http://pga.mbt.washington.edu/) and the National Institute of Environmental Health Sciences (NIEHS) Environmental Genome Project (http://www.niehs.nih.gov/) could potentially improve our power to identify more differentially expressed genes under selection in the future (Zhang and Dolan, 2008a). Finally, there are some limitations of our model using the HapMap LCLs. It should be noted that the CEU samples were collected approximately decades earlier than the YRI samples. Therefore, the collection-time difference could be a confounding factor for identifying differentially expressed genes between the CEU and YRI samples. A collection of European and Yoruba cell lines in the future will allow more accurate estimation of the variation in gene expression between these two populations, thus providing a better picture of the evolutionary history of the gene expression phenotype.

In conclusion, we report here that some differentially expressed genes between the CEU and YRI samples have been under recent positive selection. These genes were enriched in certain biological processes, molecular functions and cellular components. Our results can provide unique insights into the evolutionary history of the variation in the gene expression phenotype.

## Supplementary Material

Table S1 Evidence of selection among the genes with expression higher in the CEU samples.

Table S2 Evidence of selection among the genes with expression higher in the YRI samples.

## Figures and Tables

**Table 1:** 27 differentially expressed genes between the CEU and YRI samples are under recent positive selection.

Affymetrix Transcript Cluster ID^a^	Gene Symbol	Cytoband	P (CEU)^b^	P (YRI)^b^	Population Under Selection
***Expression Higher in CEU***^c^					
3065546	*DPY19L2P2*	7q22.1	0.0014	0.55	CEU
2560141	*MRPL53*	2p13.1	0.0024	0.36	CEU
3431892	*SH2B3*	12q24	0.0061	0.55	CEU
3226340	*PTGES2*	9q34.11	0.014	0.31	CEU
2394626	*ACOT7*	1p36.31-p36.11	0.028	0.41	CEU
2391302	*CENTB5*	1p36.33	0.030	0.64	CEU
3062868	*BAIAP2L1*	7q21.3	0.045	0.55	CEU
2672016	*FYCO1*	3p21.31	0.53	0.0098	YRI
2829171	*TCF7*	5q31.1	0.45	0.031	YRI
3230811	*DPP7*	9q34.3	0.63	0.035	YRI
3820727	*QTRT1*	19p13.3	0.79	0.038	YRI
3677752	*TRAP1*	16p13.3	0.45	0.044	YRI
***Expression Higher in YRI***^c^					
2517408	*AGPS*	2q31.2	0.00088	0.27	CEU
3568108	*SGPP1*	14q23.2	0.0056	0.077	CEU
2405893	*C1orf212*	1p34.3	0.019	0.55	CEU
3727712	*PCTP*	17q21-q24	0.023	0.077	CEU
3014855	*ZKSCAN5*	7q22	0.030	0.091	CEU
3949017	*FLJ20699*	22q13	0.037	0.36	CEU
3249738	*HNRPH3*	10q22	0.037	0.041	CEU/YRI
3464000	*CCDC59*	12q21.31	0.14	0.0010	YRI
3933817	*WDR4*	21q22.3	0.19	0.0048	YRI
3515009	*VPS36*	13q14.3	0.79	0.0098	YRI
3884324	*CTNNBL1*	20q11.23-q12	0.79	0.015	YRI
3755862	*IKZF3*	17q21	0.63	0.021	YRI
2328868	*HDAC1*	1p34	0.34	0.025	YRI
2495881	*EIF5B*	2q11.2	0.53	0.028	YRI
2737069	*METAP1*	4q23	0.29	0.038	YRI

a Affymetrix GeneChip® Human Exon 1.0 ST Array.

b empirical p value reported by Happlotter (Voight et al. 2006).

c expression profile (Zhang et al. 2008a).

**Table 2:** Enriched Gene Ontology terms among the genes under selection (at least 2 genes).

GO Category	GO Term	P	Corrected P^a^	Gene Symbol
Biological Progress	transcription	0.010	0.11	*CCDC59*, *VPS36*, *TCF7*, *ZKSCAN5*, *IKZF3*, *HDAC1*
	regulation of transcription from RNA polymerase II promoter	0.014	0.12	*TCF7*, *IKZF3*
	apoptosis	0.027	0.20	*SGPP1*, *CTNNBL1*
Molecular Function	lipid binding	0.0057	0.032	*VPS36*, *PCTP*
	binding	0.020	0.077	*FLJ20699*, *CTNNBL1*
	metal ion binding	0.035	0.11	*ZKSCAN5*, *QTRT1*, *FYCO1*, *IKZF3*, *METAP1*, *CENTB5*
	transcription factor activity	0.048	0.15	*ZKSCAN5*, *IKZF3*, *HDAC1*
Cellular Component	lysosome	0.0031	0.092	*VPS36*, *DPP7*
	mitochondrion	0.016	0.11	*AGPS*, *MRPL53*, *TRAP1*, *PTGES2*, *ACOT7*

a Corrected p value after BH correction (Benjamini and Hochberg 1995).
